# Plasma Membrane Redox Failure Links COVID-19 Metabolic Stress to Ferroptotic Neurodegeneration

**DOI:** 10.3390/antiox15050572

**Published:** 2026-05-01

**Authors:** Jaewang Lee, Hyosin Hwang, Dong-Hoon Hyun

**Affiliations:** 1 LogSynk Ltd., Seoul 06153, Republic of Korea; 2 Department of Life Science, Ewha Womans University, Seoul 03760, Republic of Korea

**Keywords:** ferroptosis, plasma membrane redox system (PMRS), COVID-19, lipid peroxidation, NAD^+^ metabolism

## Abstract

Oxidative stress and redox imbalance are central features of both age-related neurodegenerative disorders and the persistent neurological sequelae of coronavirus disease 2019. Increasing evidence suggests that severe acute respiratory syndrome coronavirus 2 (SARS-CoV-2) infection disrupts neuronal redox homeostasis via mitochondrial dysfunction, iron dysregulation, inflammatory signaling, and the depletion of pyridine nucleotide pools. In that context, ferroptosis provides a unifying mechanistic framework linking lipid peroxidation to progressive neuronal injury. This review proposes that neuronal vulnerability might depend not only on the oxidative burden itself but also on the failure of membrane-localized antioxidant defenses. Particular emphasis is placed on the plasma membrane redox system (PMRS), a membrane-associated quinone-reducing network that can support coenzyme Q redox cycling and constrain lipid radical propagation at the plasma membrane. Unlike canonical ferroptosis defense systems that rely predominantly on NADPH, components of the PMRS, particularly cytochrome b5 reductase, can also use NADH, conferring partial metabolic flexibility in conditions of redox stress. We further discuss how SARS-CoV-2-induced NAD^+^ depletion might progressively destabilize this membrane-proximal defense architecture, potentially lowering the ferroptotic threshold of vulnerable neurons. Finally, we outline therapeutic strategies that might reinforce PMRS-dependent membrane redox control through NRF2 activation, NAD^+^ restoration, coenzyme Q-centered interventions, and modulation of iron-catalyzed lipid oxidation.

## 1. Introduction

Oxidative stress and redox imbalance are central drivers of neuronal vulnerability in both age-related neurodegenerative disorders (NDs) and the emerging neurological sequelae of severe acute respiratory syndrome coronavirus 2 (SARS-CoV-2) infection [1,2,3]. Alzheimer’s disease, Parkinson’s disease, and amyotrophic lateral sclerosis are all characterized by chronic oxidative injury, sustained neuroinflammation, and progressive disruption of cellular antioxidant capacity [1,4,5]. Accumulating clinical, biochemical, and neuroimaging evidence indicates that survivors of coronavirus disease 2019 (COVID-19) can develop persistent cognitive impairment and structural brain alterations that resemble features of those redox-associated pathologies [6,7,8]. Those observations suggest that viral infection might accelerate or unmask redox vulnerabilities intrinsic to the aging nervous system.

At the molecular level, these apparently distinct conditions converge on lipid peroxidation as a critical determinant of neuronal injury [9,10,11]. Neurons are particularly susceptible to oxidative damage because of their high oxygen consumption and plasma membranes enriched in polyunsaturated fatty acids (PUFAs), which serve as preferred substrates for radical-driven chain reactions [9,10,12]. When lipid peroxidation exceeds the buffering capacity of endogenous antioxidant systems, it culminates in ferroptosis, a regulated form of iron-dependent cell death that links redox imbalance, lipid metabolism, and iron homeostasis [13,14,15]. Therefore, ferroptosis provides a useful mechanistic framework for understanding how metabolic stress can be translated into progressive neuronal loss.

In physiological conditions, neuronal membranes are protected by multiple, partially overlapping antioxidant defense systems. These include the glutathione (GSH)-dependent glutathione peroxidase 4 (GPX4) pathway, which detoxifies phospholipid hydroperoxides [16,17,18], and the ferroptosis suppressor protein 1 (FSP1)–coenzyme Q (CoQ; ubiquinol) axis, which limits lipid radical propagation at the plasma membrane [19,20,21]. The coexistence of these systems reflects the biological need for antioxidant redundancy in cells that must preserve membrane integrity across large membrane surfaces and extended physiological time scales. However, this redundancy is conditional rather than absolute, and it becomes increasingly constrained during sustained inflammatory and metabolic stress.

SARS-CoV-2 infection imposes precisely such constraints. Virus-induced systemic inflammation disrupts iron metabolism, thereby favoring Fenton chemistry and amplifying lipid peroxidation [22,23]. That cytokine-rich inflammatory milieu—characterized by elevated interleukin (IL)-6 and tumor necrosis factor alpha—can suppress the cystine/glutamate antiporter system Xc^−^ (xCT), promoting GSH depletion and compromising GPX4-dependent detoxification [24]. Simultaneously, virus-associated mitochondrial dysfunction and oxidative DNA damage activate NAD^+^-consuming pathways, which leads to NAD^+^ depletion and the contraction of cellular pyridine nucleotide pools [25,26,27]. This metabolic exhaustion weakens NAD(P)H-dependent antioxidant reactions and impairs multiple ferroptosis defense mechanisms.

Antioxidant failure should not be interpreted as the collapse of a single pathway but as destabilization of an integrated membrane defense network. Although GPX4 and FSP1 are major ferroptosis-protective layers, their activity is constrained by cysteine availability, GSH status, and the NADPH supply [18,28,29]. Those metabolic dependencies become particularly limiting during SARS-CoV-2 infection, when mitochondrial dysfunction, poly(ADP-ribose) polymerase 1 (PARP-1) activation, and inflammatory signaling reduce cellular redox flexibility [26,27,30]. The plasma membrane redox system (PMRS) comprises enzymes such as NAD(P)H:quinone oxidoreductase 1 (NQO1) and cytochrome b5 reductase (CYB5R), which are localized at the plasma membrane and intracellular membrane compartment and catalyze quinone reduction reactions that support CoQ redox cycling and membrane-associated antioxidant defense. This system is therefore of particular interest because it directly supports the regeneration of reduced CoQ at the membrane interface and limits lipid radical propagation [31,32,33]. Of note, CYB5R can use NADH as an electron donor, providing metabolic flexibility when NADPH availability becomes restricted [33]. This property suggests that the PMRS might function not merely as a redundant auxiliary pathway but also as a membrane-proximal contributor to the ferroptotic threshold during virus-induced metabolic stress.

**Figure 1:**
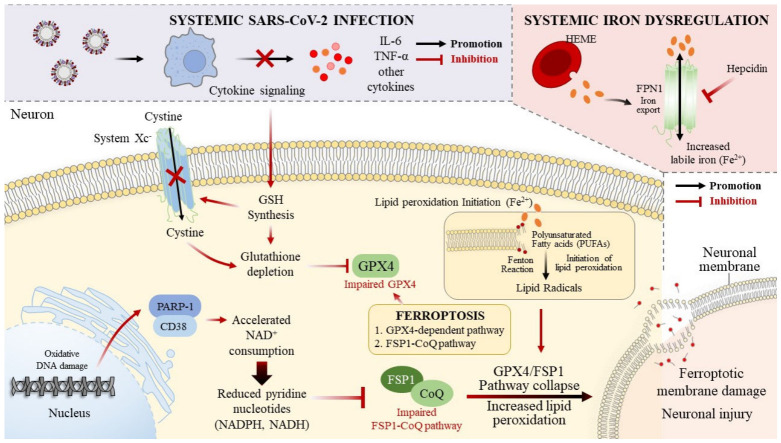
**Metabolic and redox mechanisms linking SARS-CoV-2 infection to ferroptotic neuronal vulnerability.** SARS-CoV-2 infection disrupts neuronal redox homeostasis through systemic inflammation, iron dysregulation, and mitochondrial dysfunction. Cytokine signaling suppresses cystine uptake via system Xc^−^, leading to glutathione depletion and impaired GPX4-dependent detoxification of phospholipid hydroperoxides. Concurrently, oxidative DNA damage activates PARP-1 and CD38, accelerating NAD^+^ consumption and reducing pyridine nucleotide availability. Those metabolic changes weaken the GPX4 and FSP1–CoQ ferroptosis defense systems. Increased labile iron further promotes Fenton chemistry and initiates lipid peroxidation in polyunsaturated neuronal membranes. When the antioxidant buffering capacity is exceeded, lipid radical propagation might lead to ferroptotic membrane damage and neuronal injury. CD38, cluster of differentiation 38; CoQ, coenzyme Q (ubiquinone); FPN1, ferroportin 1; FSP1, ferroptosis suppressor protein 1; GPX4, glutathione peroxidase 4; IL-6, interleukin-6; NAD^+^, oxidized nicotinamide adenine dinucleotide; NADH, reduced nicotinamide adenine dinucleotide; NADPH, reduced nicotinamide adenine dinucleotide phosphate; PARP-1, poly(ADP-ribose) polymerase 1; SARS-CoV-2, severe acute respiratory syndrome coronavirus 2; system Xc^−^, cystine/glutamate antiporter; TNF-α, tumor necrosis factor-α.

In this review, we examine how virus-induced metabolic stress, iron dysregulation, and nuclear factor erythroid 2-related factor 2 (NRF2) suppression converge to destabilize the neuronal antioxidant network, with particular emphasis on the PMRS. We propose that the transition from reversible oxidative stress to irreversible ferroptotic neurodegeneration might depend not only on the magnitude of the oxidative burden but also on the failure of membrane-localized redox control (Figure 1).

From this perspective, the PMRS emerges as a plausible mechanistic node linking SARS-CoV-2-associated metabolic exhaustion to neuronal membrane vulnerability, making it a hypothesis-generating target for potential mechanism-based antioxidant interventions. This review integrates emerging evidence on ferroptosis, metabolic stress, and membrane redox control to highlight the PMRS as a potential contributor to neuronal ferroptotic vulnerability in COVID-19-associated neurologic injury.

## 2. Ferroptosis and Neuroinflammation: An Oxidative Feedback Loop

The pathogenesis of NDs and the neurological sequelae of COVID-19 increasingly appears to be driven by a lethal intersection between iron-dependent lipid peroxidation and chronic immune activation [13,15,34]. Ferroptosis is biochemically defined by iron-catalyzed lipid peroxidation and requires redox-active iron, specific PUFA-containing phospholipids, and the failure of lipid-peroxide repair mechanisms [13,14,34]. A defining feature of ferroptotic injury is self-propagating lipid peroxidation within biological membranes [9,11]. Polyunsaturated phospholipids contain bis-allylic hydrogen atoms that are highly susceptible to abstraction by reactive oxygen species (ROS). Once formed, lipid radicals react rapidly with molecular oxygen to generate lipid peroxyl radicals, which then attack adjacent phospholipids and propagate chain reactions across the membrane bilayer [9]. This autocatalytic process can spread rapidly along neuronal membranes, particularly within synaptic terminals where lipid turnover is high and antioxidant diffusion is low.

Neuronal membranes are especially vulnerable because they are enriched in long-chain PUFAs such as arachidonic acid (AA) and docosahexaenoic acid (DHA) [35]. These lipids confer the structural flexibility required for synaptic plasticity, but they also increase susceptibility to oxidative attack. Vulnerability is further amplified by the high surface-area-to-volume ratio of neuronal processes, as extensive axonal and dendritic membranes must maintain redox stability over long distances from the cell body. Consequently, even moderate increases in ROS can initiate localized lipid peroxidation that subsequently propagates along neuronal membranes.

The propagation phase of lipid peroxidation marks the critical transition from reversible oxidative stress to irreversible ferroptotic damage [9,11]. In the early stages, antioxidant systems can intercept lipid peroxyl radicals and terminate chain reactions [9]. Once propagation exceeds that buffering capacity, however, lipid hydroperoxides accumulate and destabilize membrane integrity [34]. Secondary breakdown products such as malondialdehyde (MDA) and 4-hydroxynonenal (4-HNE) exacerbate injury by modifying proteins and nucleic acids [12,36]. Because these aldehydic products can diffuse away from their site of origin, they also extend oxidative stress to neighboring cells and amplify neuroinflammatory signaling within the central nervous system (CNS) [11].

Taken together, these processes position ferroptotic lipid peroxidation not only as a terminal mechanism of neuronal injury but also as a catalytic driver of neuroinflammatory signaling, thereby establishing a self-reinforcing oxidative loop that places exceptional demand on membrane-localized antioxidant systems.

Within that framework, the spatial organization of antioxidant defenses becomes a decisive determinant of cellular susceptibility to ferroptosis [14,15]. Although cytosolic antioxidant systems neutralize diffusible ROS, lipid radical initiation and propagation occur primarily within the membrane bilayer [9]. Effective protection therefore requires antioxidant mechanisms that operate directly at the lipid interface. The PMRS might contribute to this role by regenerating reduced ubiquinol pools at the membrane surface, enabling the rapid quenching of lipid peroxyl radicals before chain propagation becomes self-sustaining [31,32,33].

In the CNS, where neurons combine a distinct lipid composition with high metabolic oxygen consumption, ferroptosis is a major oxidative execution pathway that contributes to neuronal loss [37,38]. Iron is essential for myelin synthesis and neurotransmission, but its concentration must be tightly regulated by ferritin sequestration [22,39]. Under the stress of COVID-19 or chronic neurodegeneration, ferritinophagy—the ferritin degradation process mediated by nuclear receptor coactivator 4—increases the labile iron pool and fuels Fenton chemistry at neuronal plasma membranes [34,40]. In parallel, enzymatic lipid remodeling by acyl-CoA synthetase long-chain family member 4 and lysophosphatidylcholine acyltransferase 3 enriches the membranes with AA- and DHA-containing phospholipids, further increasing their susceptibility to lipid peroxidation [41,42,43]. Together, those changes create an environment permissive to ferroptotic initiation.

Emerging evidence suggests that ferroptotic lipid damage might also have immunogenic effects within the CNS. Oxidized lipid species and reactive aldehydes—including MDA and 4-HNE—released upon membrane rupture act as damage-associated molecular patterns that activate pattern recognition receptors on microglia and astrocytes and promote inflammasome activation via nucleotide-binding oligomerization domain-like receptor family pyrin domain-containing 3 [4,5]. The resulting secretion of pro-inflammatory cytokines, including IL-1β and IL-6, exacerbates iron dysregulation and suppresses neuronal antioxidant defenses, thereby reinforcing the ferroptotic cascade [5,34]. This feed-forward loop could help explain the persistence of neuroinflammation in long-COVID patients, despite viral clearance [7,8]. Within this setting, the burden on membrane-localized antioxidant systems becomes particularly severe. Because lipid peroxidation is initiated and propagated at the plasma membrane, membrane-proximal redox control becomes a critical determinant of neuronal survival. The PMRS operates at this interface, maintaining membrane-associated ubiquinol pools that intercept lipid peroxyl radicals, thereby limiting chain propagation and modulating the inflammatory consequences of ferroptotic injury.

## 3. The PMRS as a Metabolically Flexible Antioxidant Defense

**Figure 2:**
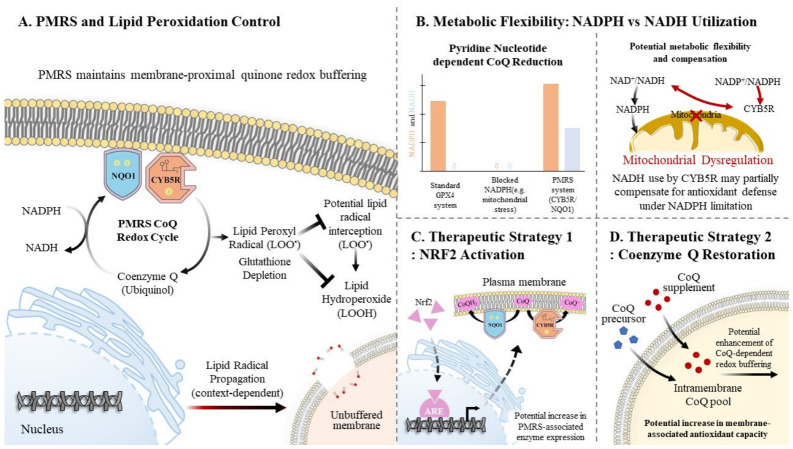
**Conceptual model of PMRS-associated membrane redox control and its potential contribution to ferroptotic vulnerability.** (**A**) The plasma membrane redox system (PMRS), comprising membrane-associated reductases such as NAD(P)H:quinone oxidoreductase 1 (NQO1) and cytochrome b5 reductase (CYB5R), contributes to quinone redox cycling at the plasma membrane. By regenerating reduced coenzyme Q (CoQ; ubiquinol), the PMRS might support the interception of lipid peroxyl radicals and thereby influence the propagation of membrane lipid peroxidation. (**B**) In contrast to several other ferroptosis defense pathways that depend predominantly on NADPH, CYB5R can use NADH as an electron donor. This feature suggests potential metabolic flexibility during mitochondrial dysfunction or NADPH limitation, which could partially restore the membrane redox buffering capacity when canonical antioxidant systems are constrained. (**C**,**D**) The therapeutic strategies illustrated (NRF2 activation, NAD^+^ restoration, and CoQ-centered approaches) are conceptual and intended to represent potential mechanisms that might influence the membrane redox balance. These interventions are likely to act indirectly through broad metabolic and redox pathways, rather than through specific targeting of the PMRS. In this hypothetical framework, disruption of pyridine nucleotide availability, as observed in SARS-CoV-2-associated metabolic stress, could progressively weaken the GPX4-, FSP1-, and PMRS-related antioxidant systems, thereby increasing susceptibility to ferroptotic membrane damage. However, the relative contribution of the PMRS to ferroptosis regulation, particularly in the context of neuronal diseases or COVID-19, remains to be directly established. ARE, antioxidant response element; CoQ, coenzyme Q (ubiquinone); FSP1, ferroptosis suppressor protein 1; GPX4, glutathione peroxidase 4; NAD^+^, oxidized nicotinamide adenine dinucleotide; NADH, reduced nicotinamide adenine dinucleotide; NADPH, reduced nicotinamide adenine dinucleotide phosphate; NRF2, nuclear factor erythroid 2–related factor 2; SARS-CoV-2, severe acute respiratory syndrome coronavirus 2.

Among the antioxidant systems that constrain lipid peroxidation, the PMRS occupies a distinct functional niche (Figure 2) [31,32,44]. The major ferroptosis defense systems and their metabolic dependencies are summarized in Table 1. Cellular protection against ferroptosis is mediated not by a single antioxidant pathway but by a coordinated network of complementary defense systems operating across subcellular compartments [14,15,34].

At the core of this network lies the GSH–GPX4 axis, which enzymatically reduces phospholipid hydroperoxides to non-toxic alcohols [16,17,18]. This pathway is the canonical ferroptosis suppressor and is essential for maintaining lipid redox balance in physiological conditions [13,15]. However, GPX4 activity depends on sufficient GSH availability and continuous NADPH regeneration, both of which become compromised during inflammatory or metabolic stress [15,18,28].

To provide redundancy, cells have additional mechanisms that suppress lipid radical propagation independently of GSH. One such pathway is the FSP1–CoQ system, which operates at the plasma membrane [19]. FSP1 reduces ubiquinone to ubiquinol using NADPH as an electron donor, and the resulting ubiquinol functions as a potent radical-trapping antioxidant that intercepts lipid peroxyl radicals and terminates chain reactions [19,45]. This pathway functions in parallel with GPX4 and can partially compensate for GSH depletion.

A further layer of protection is provided by mitochondrial dihydroorotate dehydrogenase, which contributes to the maintenance of reduced CoQ pools within mitochondria and thereby suppresses lipid peroxidation in mitochondrial membranes [29]. Together, these compartmentalized ferroptosis defense systems form a hierarchical redox architecture that stabilizes lipid membranes across cellular compartments [15,34].

Within that network, the PMRS occupies a distinctive functional position. Unlike GPX4 or FSP1, which rely strictly on NADPH, components of the PMRS—particularly CYB5R—can use NADH as an electron donor [31,33]. This metabolic flexibility enables the PMRS to sustain the membrane redox balance even when the NADPH supply becomes inadequate [31,32].

However, this flexibility should be interpreted as a transient and quantitatively constrained adaptation. The extent to which NADH can sustain PMRS-associated redox activity is likely limited by substrate availability, enzyme kinetics, and competing metabolic demands, and its capacity to compensate for NADPH deficiency over extended periods remains to be determined.

During metabolic stress, such as viral infection or mitochondrial dysfunction, this alternative electron source can allow the PMRS to transiently maintain antioxidant protection when other systems are compromised.

Functionally, the PMRS operates as a membrane-proximal redox buffering system that preserves reduced CoQ pools at the plasma membrane [31,32,45]. This localization is critical because lipid radical initiation and propagation occur primarily within the membrane bilayer [9]. By maintaining ubiquinol availability at the lipid interface, the PMRS helps determine the threshold at which lipid peroxidation shifts from reversible oxidation to self-sustaining ferroptotic propagation. The PMRS consists of membrane-associated reductases and electron carriers that maintain quinone redox cycling at the cell surface [31,32]. Within this system, NQO1 and CYB5R play complementary roles [33,46]. NQO1 catalyzes a two-electron reduction in quinones, directly generating stable hydroquinones while preventing the formation of pro-oxidant semiquinone intermediates [46]. Through this mechanism, NQO1 contributes to the regeneration of ubiquinol, a key lipophilic radical-trapping antioxidant [45].

Transcriptional control by NRF2 further integrates PMRS activity into global redox regulation. NRF2 coordinates inducible antioxidant responses, including the expression of NQO1 and xCT, during oxidative stress [47,48]. SARS-CoV-2-mediated suppression of NRF2 signaling might therefore restrict compensatory upregulation of PMRS components, diminishing this membrane-associated antioxidant layer in parallel with canonical ferroptosis defense systems [49,50]. Experimental evidence from viral infection and inflammatory stress models supports a link between NRF2 repression and increased ferroptotic susceptibility [48,49].

From an antioxidant network perspective, the PMRS can therefore be viewed as a stress-adaptive component of the cellular redox architecture, rather than a simple auxiliary pathway. Although direct evidence linking PMRS activity to ferroptosis suppression remains sparse, its ability to regenerate membrane ubiquinol suggests a potential role in constraining lipid radical propagation. When GSH depletion limits GPX4 activity and NADPH scarcity weakens FSP1-mediated CoQ reduction, the PMRS might transiently mitigate lipid peroxidation at the membrane interface [31,32,33]. However, sustained metabolic exhaustion and transcriptional suppression can ultimately compromise this system as well, suggesting that PMRS integrity could influence the ferroptotic threshold of neurons during prolonged metabolic stress.

Notably, the PMRS should not be interpreted as replacing other plasma membrane ferroptosis defenses such as FSP1. Instead, these systems likely operate as partially overlapping redox buffers that differ in metabolic coupling and regulatory control. When NADPH availability becomes a limiting factor or transcriptional antioxidant responses are suppressed, PMRS-mediated quinone reduction might provide an additional layer of membrane redox resilience, rather than functioning as a singular or exclusive ferroptosis suppressor.

Compared with established ferroptosis defense systems such as GPX4 and FSP1, which are supported by extensive genetic and pharmacological evidence, the PMRS remains comparatively less well characterized in the context of ferroptosis. GPX4 directly reduces phospholipid hydroperoxides using GSH, and FSP1 regenerates CoQ in a NADPH-dependent manner at the plasma membrane, both with well-defined roles in suppressing ferroptotic cell death. In contrast, PMRS-associated components such as NQO1 and CYB5R are primarily supported by biochemical and indirect evidence, and their contribution to ferroptosis regulation remains to be established. This distinction underscores that the PMRS is presented here as a candidate membrane-associated redox system with potential relevance, rather than as a functionally equivalent core regulator.

In this framework, the ferroptotic threshold might be determined by the balance among lipid hydroperoxide accumulation, the CoQ redox state, GPX4 activity, and the availability of reducing equivalents such as NADH and NADPH. Experimentally, this threshold could be approximated through combined measurements of lipid peroxidation dynamics, quinone redox state, and enzymatic antioxidant activity, but precise quantitative definitions remain to be established.

Despite the current lack of precise quantitative thresholds, the capacity of NADH to sustain PMRS function is likely limited over time and might become insufficient during prolonged metabolic stress or sustained NAD^+^ depletion.

## 4. SARS-CoV-2, NAD^+^ Depletion, and Metabolic Constraints on Antioxidant Defenses

The neurological manifestations of COVID-19 largely reflect the disruption of neuronal bioenergetics, rather than direct viral cytotoxicity. SARS-CoV-2 infection induces mitochondrial fragmentation, impairs oxidative phosphorylation, and increases mitochondrial ROS production, collectively decreasing cellular reducing capacity [27,30,51]. Those alterations sensitize neurons to ferroptosis by constraining the metabolic infrastructure required to sustain antioxidant defenses [15,34].

A defining metabolic feature of COVID-19 is rapid depletion of NAD^+^, often described as an “NAD^+^ drought” [25,26,27]. Oxidative DNA damage activates PARP-1, which consumes NAD^+^ during DNA repair [25,26]. In parallel, inflammatory signaling induces cluster of differentiation 38 (CD38) expression, further accelerating NAD^+^ turnover [25]. Together, these pathways progressively drain the cellular pyridine nucleotide pools required to regenerate NADH and NADPH.

The loss of pyridine nucleotide availability has immediate consequences for ferroptosis defense systems. Reduced NADPH availability compromises GPX4 activity and FSP1-mediated CoQ reduction [19,28], and declining NAD^+^ limits the NADH production required for CYB5R-dependent PMRS functioning [33]. In this context, NAD^+^ depletion could represent an upstream metabolic event that helps to convert mitochondrial dysfunction into plasma membrane redox vulnerability. Ferroptosis might therefore emerge not simply from increased oxidative stress but from progressive failure of antioxidant defenses that are spatially organized at the membrane interface [14,15,34].

Beyond global depletion, disruption of pyridine nucleotide compartmentalization further weakens cellular redox resilience. Mitochondrial dysfunction alters the balance between cytosolic and mitochondrial NAD(H) pools, impairing redox shuttles such as the malate–aspartate and glycerol-3-phosphate systems that normally balance reducing equivalents between compartments [27,51]. Inflammatory inhibition of the pentose phosphate pathway also restricts de novo NADPH generation, partially decoupling cytosolic redox buffering from mitochondrial metabolism [26]. These alterations promote functional redox isolation between organelles and reduce the efficiency with which cells redistribute reducing equivalents during metabolic stress.

Within this altered metabolic landscape, the PMRS might temporarily preserve membrane redox control. Because CYB5R can use NADH derived from glycolysis, the PMRS can exploit residual cytosolic reducing equivalents even when mitochondrial metabolism is compromised [33]. This possibility should be interpreted as a transient metabolic contingency, rather than a universally preserved pathway. During early or moderate metabolic stress, compensatory glycolytic flux might maintain partial NADH production, even as mitochondrial oxidative phosphorylation declines. However, in the advanced metabolic collapse associated with severe systemic hypoxia or prolonged inflammatory stress, both NADH and NADPH regeneration can become progressively constrained, ultimately compromising PMRS function, as well as that of the other ferroptosis defense systems.

This metabolic hierarchy introduces a temporal dimension to ferroptotic vulnerability. Initially, glycolysis-derived NADH can support PMRS-mediated electron transfer and maintain membrane redox buffering, despite rising oxidative pressure. However, sustained activation of NAD^+^-consuming pathways progressively erodes pyridine nucleotide availability [25,26,27]. As NAD^+^ depletion intensifies, the regeneration of both NADH and NADPH becomes increasingly constrained, leading to simultaneous weakening of GPX4-, FSP1-, and PMRS-dependent antioxidant defenses [19,28,33]. Once that metabolic threshold is crossed, lipid peroxidation can propagate unchecked across neuronal membranes, marking the transition from adaptive oxidative stress to irreversible ferroptotic injury [14,34].

These processes might develop over time, with early metabolic stress potentially representing a reversible state, whereas sustained NAD^+^ depletion and lipid peroxidation might drive a transition toward irreversible ferroptotic injury. Such temporal progression could contribute to the distinction between acute neurological symptoms and persistent post-COVID sequelae.

Clinically, this temporal distinction may be reflected in the heterogeneity of post-COVID neurological symptoms. Early-phase manifestations such as fatigue, impaired attention, and so-called “brain fog” may predominantly arise from reversible metabolic dysfunction and redox imbalance. In contrast, persistent cognitive impairment and memory deficits are more likely to reflect cumulative oxidative damage and potential ferroptotic neuronal loss. Although direct causal relationships remain to be established, this framework suggests that the contribution of ferroptosis may increase over time as metabolic stress becomes sustained and membrane redox control progressively fails.

Taking all the evidence together, it appears that SARS-CoV-2-induced NAD^+^ depletion progressively destabilizes the pyridine-nucleotide-dependent antioxidant network that maintains membrane redox balance. In this framework, ferroptotic vulnerability arises not only from increased ROS production but also from exhaustion of the metabolic systems required to sustain membrane-localized antioxidant protection. The PMRS therefore emerges as a metabolically sensitive node that might link cellular energy metabolism to the preservation of neuronal membrane integrity during virus-induced stress.

In addition to neuronal vulnerability, cell-type-specific responses might further shape ferroptotic susceptibility in the CNS. Astrocytes and microglia play critical roles in redox homeostasis, iron handling, and inflammatory signaling, and they might differentially influence lipid peroxidation dynamics and antioxidant capacity in neighboring neurons. However, the contribution of these non-neuronal cell types to PMRS-associated membrane redox regulation remains to be defined.

Interindividual variability in metabolic state, age, comorbidities, and genetic background could further influence susceptibility to membrane redox imbalance and ferroptotic injury. Such variability could contribute to the heterogeneous neurological outcomes observed in COVID-19, although direct evidence linking those factors to PMRS function remains inadequate.

## 5. Therapeutic Strategies Targeting the PMRS

The identification of the PMRS as a determinant of membrane redox stability reframes antioxidant intervention strategies for COVID-19-related neurodegeneration [31,32,33]. Conventional antioxidant supplementation has shown limited efficacy in NDs, likely because it does not adequately address the enzymatic, spatial, and metabolic constraints that govern lipid peroxidation [3,38]. In contrast, PMRS-oriented strategies would aim to preserve membrane-localized control of lipid radical propagation and thus intervene closer to the site at which ferroptotic injury is initiated [14,15]. However, existing approaches primarily act on broad redox and metabolic pathways, rather than directly targeting the PMRS. Therapeutic approaches intended to stabilize membrane-associated redox processes might help to maintain the membrane redox balance and raise the threshold for ferroptotic lipid peroxidation in metabolically stressed neurons. Representative therapeutic strategies targeting PMRS-dependent membrane redox control are summarized in Table 2.

One major therapeutic entry point involves reinforcing the transcriptional programs that sustain PMRS capacity. Activation of the NRF2 pathway is a central strategy in this regard [47,48]. Small-molecule NRF2 activators such as sulforaphane and dimethyl fumarate disrupt Kelch-like ECH-associated protein 1-dependent degradation of NRF2, thereby inducing the expression of antioxidant and detoxification genes such as NQO1 and xCT, along with other redox-protective pathways [48,49,50]. In the present framework, NRF2 activation is important not merely because it increases overall antioxidant tone but because it can restore membrane-proximal quinone reduction capacity through NQO1 induction [46,47]. The therapeutic objective is therefore not indiscriminate radical scavenging but the restoration of enzymatic control over CoQ redox cycling at the membrane interface.

In other words, the protective role of NQO1 is context-dependent. Although NQO1 catalyzes a two-electron reduction in quinones that generally prevents the formation of unstable semiquinone intermediates, excessive induction in conditions of inadequate electron acceptor availability or quinone overload can theoretically promote redox cycling of reduced quinone species. In this framework, the therapeutic induction of NQO1 is thus conceptualized not as maximal upregulation but as restoration of the physiological membrane CoQ redox buffering capacity, consistent with a hormetic redox resuscitation model, rather than supraphysiological enzyme activation.

A second therapeutic axis involves preservation of the metabolic substrate supply required for PMRS function. Because CYB5R can use NADH as an electron donor and broad PMRS activity depends on pyridine nucleotide availability, the restoration of intracellular NAD^+^ pools is a logical strategy for maintaining membrane redox resilience [26,33]. Supplementation with nicotinamide riboside or nicotinamide mononucleotide might help to replenish NAD^+^, sustain NADH/NADPH regeneration, and thereby support membrane-associated electron transfer during virus-induced metabolic stress [26,54]. In settings characterized by severe PARP-1 overactivation, however, precursor supplementation alone could be insufficient, and combined approaches that limit excessive NAD^+^ consumption might be required [25,26]. From this perspective, NAD^+^ restoration should be viewed not only as a bioenergetic intervention but also as a strategy to preserve the reducing infrastructure required for PMRS-dependent membrane protection.

A third therapeutic approach focuses on reinforcing membrane CoQ redox buffering. Because ubiquinol functions as a lipophilic radical-trapping antioxidant, maintaining a reducible CoQ pool at the plasma membrane might help to interrupt lipid peroxyl radical propagation before chain reactions become self-sustaining [45]. Ubiquinol supplementation therefore has conceptual appeal within this framework, particularly when it is coupled with enzymatic systems that can regenerate the reduced quinone state [31,32]; however, its efficacy in neuronal contexts remains uncertain. Experimental radical-trapping agents such as ferrostatin-1 and liproxstatin-1 demonstrate that selective stabilization of the membrane lipid redox balance can suppress ferroptotic execution in experimental systems [57,58]; however, their clinical applicability remains limited, and they should be interpreted as proof-of-concept tools rather than direct therapeutic strategies. Although those compounds are currently research tools, they illustrate the principle that effective antioxidant therapy could require intervention at the level of membrane lipid chemistry, rather than bulk ROS neutralization.

Because ferroptosis is also driven by iron-catalyzed initiation chemistry, the modulation of iron handling is a complementary therapeutic dimension [22,56]. Brain-permeable iron chelators could reduce the catalytic pressure driving phospholipid oxidation and thereby lower the burden placed on PMRS-dependent antioxidant systems [22]. Mechanistically, this approach does not directly strengthen the PMRS, but it preserves its functional reserve by reducing the rate at which lipid radical propagation is initiated. Such strategies could be particularly relevant in COVID-19-associated states characterized by ferritin dysregulation, inflammatory iron redistribution, and enhanced ferritinophagy.

Additional compounds could modulate the PMRS more directly. Emerging evidence suggests that 4-hydroxycinnamic acid (4-HCA) can attenuate neuronal oxidative injury, partly by inducing the expression of NQO1 and CYB5R [53], which raises the possibility that certain neurohormetic agents could strengthen the PMRS as an adaptive membrane defense program [52]. It should be noted that the precise mechanism of action of 4-HCA remains incompletely defined. In addition to potential NRF2-mediated induction of antioxidant enzymes, hydroxycinnamic acid derivatives might also have modest radical-scavenging activity or influence cellular iron handling. Within the conceptual framework proposed here, however, the primary relevance of 4-HCA lies in its potential to induce endogenous membrane-protective antioxidant systems, rather than its ability to act as a direct chemical antioxidant.

Although this evidence remains preliminary, it supports a broad conceptual strategy in which controlled redox signaling induces endogenous membrane-protective systems, rather than relying exclusively on exogenous antioxidants.

These interventions can thus be organized into a mechanistic hierarchy: NRF2 activation might enhance antioxidant capacity at the transcriptional level; NAD^+^ restoration could preserve the metabolic reducing power required for redox processes; CoQ-centered approaches might reinforce radical-trapping capacity at the membrane surface; and iron modulation could reduce upstream oxidative pressure. This layered strategy aligns more closely with the present model than conventional antioxidant supplementation because it targets the enzymatic and metabolic architecture of membrane redox control. Future therapeutic development should therefore focus not only on whether oxidative stress is present but also on which membrane-localized control nodes fail first, in what metabolic conditions they fail, and in which patient populations such failures are most likely to become clinically significant.

These observations highlight the principle that effective ferroptosis modulation might require intervention at the level of membrane lipid chemistry, although translation into clinically viable strategies remains an open challenge.

In addition, the clinical feasibility of these interventions remains uncertain, as factors such as dosing, pharmacokinetics, and tissue-specific delivery could substantially influence their effectiveness in human neurological conditions.

## 6. Perspectives and Conclusions

The neurological legacy of the COVID-19 pandemic highlights a fundamental vulnerability of the aging brain: a limited capacity to preserve membrane redox stability during sustained metabolic and inflammatory stress. In this context, ferroptosis provides a unifying mechanistic framework linking virus-associated redox imbalance, iron dysregulation, lipid peroxidation, and progressive neuronal dysfunction. The present synthesis further suggests that the PMRS might occupy a critical position within this framework because it operates directly at the membrane interface, where lipid radical propagation is initiated and ferroptotic injury becomes structurally consequential.

A central implication of this model is that neuronal susceptibility might depend not only on the total oxidative burden but also on the integrity of spatially organized antioxidant defenses. The transition from reversible oxidative stress to irreversible ferroptotic injury might therefore occur when membrane-localized redox control can no longer sustain reduced CoQ pools, terminate lipid peroxyl radical propagation, or compensate for broad metabolic exhaustion. From this perspective, the PMRS might integrate the metabolic state, transcriptional regulation, and lipid antioxidant defense into a membrane-proximal determinant of neuronal resilience.

Taken together, this framework suggests that virus-induced metabolic stress might lower the ferroptotic threshold of neurons by progressively eroding membrane-localized redox control systems.

At the same time, this interpretation should be considered with appropriate caution. Direct evidence linking PMRS failure to post-COVID neurodegeneration in human neural tissue remains sparse, and several mechanistic connections discussed here are currently supported more strongly by biochemical inference, cellular models, or related neurodegenerative contexts than by longitudinal human studies. Accordingly, the PMRS should not yet be regarded as a definitive causal driver, but rather as a plausible and testable mechanistic node that might help explain how virus-induced metabolic stress lowers the ferroptotic threshold of vulnerable neurons.

Significantly, the PMRS–ferroptosis axis should not be interpreted in isolation. Multiple complementary mechanisms have been implicated in COVID-19-related neuronal injury, including direct viral neuroinvasion, persistent neuroinflammation, microglial activation, vascular dysfunction, excitotoxicity, and other forms of regulated cell death such as apoptosis, pyroptosis, and necroptosis. These processes are unlikely to act as mutually exclusive pathways, but rather interact within a shared metabolic and redox landscape, where diverse forms of cellular stress converge.

Within this broader context, the PMRS–ferroptosis axis is not proposed as a dominant or exclusive mechanism. Instead, it is prioritized here as a membrane-proximal integrative node that links upstream insults—such as inflammatory signaling, vascular compromise, excitotoxic stress, and viral effects—to downstream lipid peroxidation dynamics and antioxidant capacity. This positioning provides a mechanistic framework through which multiple injury pathways may converge on a common process of membrane destabilization and neuronal vulnerability, thereby situating ferroptosis as one component within an interconnected network of cell death and stress-response mechanisms rather than a singular explanatory model.

This distinction has important implications for future research. Clinical and translational studies should aim to identify the conditions in which PMRS insufficiency becomes biologically relevant. Such efforts could include metabolic and genetic stratification of high-risk populations, assessment of NQO1- and CYB5R-related vulnerability, longitudinal monitoring of NAD^+^ status and lipid peroxidation markers, and the integration of redox biomarkers with cognitive and neuroimaging outcomes. Such approaches could help to clarify whether PMRS failure is an early adaptive bottleneck, a late executional event, or a stage-dependent process shaped by metabolic reserve.

Therapeutically, the present framework argues for a shift away from nonspecific antioxidant supplementation and toward targeted reinforcement of membrane-localized redox systems. Strategies that preserve NAD^+^ availability, sustain CoQ reduction, enhance NRF2-dependent membrane defense programs, and limit iron-catalyzed lipid oxidation might offer a more mechanistically coherent route to intervention than bulk antioxidant delivery alone. If validated, this perspective could position the PMRS not merely as a biochemical curiosity but as a tractable membrane-centered target for mechanism-based antioxidant therapy in COVID-19-related neurological injury and potentially in broader ferroptosis-associated neurodegenerative diseases.

## Figures and Tables

**Table 1:** Major antioxidant systems regulating membrane lipid peroxidation.

Category	Primary Enzyme	Electron Donor	Localization	Evidence in Ferroptosis	Vulnerability in COVID-19 and NDs	Functional Role	Reference
Established ferroptosis defense systems	GPX4	GSH	Cytosol, mitochondria	Extensive (genetic and pharmacologic evidence)	GSH depletion; system Xc^−^ suppression under inflammation	Detoxification of phospholipid hydroperoxide	[16,17,18]
	FSP1–CoQ axis	NADPH	Plasma membrane (myristoylated)	Well established (independent of GPX4)	NADPH depletion under metabolic stress	Radical-trapping via CoQ reduction	[19,20,21,45]
Candidate membrane-associated redox systems	NQO1	NADH/NADPH	Cytosol, membrane associated	Limited/indirect	NRF2 suppression; transcriptional dysregulation	Two-electron quinone reduction; supports CoQ redox cycling	[46,47,48]
	CYB5R	NADH	Plasma membrane, ER, mitochondria	Limited/indirect	NAD^+^ depletion; bioenergetic impairment	NADH-dependent electron transfer; potential metabolic compensation	[33]

PMRS-associated systems are included as candidate contributors based on biochemical plausibility; their direct role in ferroptosis regulation remains to be established. Abbreviations: CoQ, coenzyme Q (ubiquinone); CYB5R, cytochrome b5 reductase; ER, endoplasmic reticulum; FSP1, ferroptosis suppressor protein 1; GPX4, glutathione peroxidase 4; GSH, glutathione; NADH, nicotinamide adenine dinucleotide (reduced form); NADPH, nicotinamide adenine dinucleotide phosphate (reduced form); NQO1, NAD(P)H quinone dehydrogenase 1; NRF2, nuclear factor erythroid 2–related factor 2; PMRS, plasma membrane redox system.

**Table 2:** Interventions with the potential to influence membrane redox balance and ferroptotic vulnerability.

Category	Intervention	Proposed Mechanism	Evidence Context	Specificity to PMRS	Key Limitations	Reference
Transcriptional regulation	4-Hydroxycinnamic acid	Induction of redox enzymes (NQO1, CYB5R) and mitochondrial support	Neuronal cell models	Indirect (mechanism not fully defined)	Limited mechanistic specificity; mixed antioxidant vs. signaling effects	[52,53]
	Sulforaphane/DMF	NRF2 activation; induction of antioxidant genes	Cellular, inflammatory, and preclinical models	Indirect (broad NRF2-mediated response)	Immunomodulatory effect; not membrane-specific; safety considerations	[48,49,50]
	Calorie restriction	Hormetic activation of endogenous antioxidant pathways	Aging and metabolic models	Indirect (systemic metabolic effect)	Non-specific; translational variability	[31,32,44]
	Andrographolide	NRF2 pathway modulation; potential induction of antioxidant genes	Preclinical/natural compound studies	Indirect (broad NRF2 activation)	Mechanism not well defined; limited ferroptosis-specific evidence	[48,52]
PMRS Fuel (NADH)	NMN/NR	Restoration of NAD^+^ pools supports NADH/NADPH-dependent redox processes	Preclinical and limited human studies	Indirect (global metabolic effect)	Variable bioavailability; uncertain neuronal NAD^+^ repletion	[54,55]
Radical Quenching	Ubiquinol (CoQ_10_)	Radical trapping antioxidant; supports membrane CoQ redox cycling	Biochemical and preclinical models	Indirect (substrate-level support)	Poor bioavailability; limited BBB penetration	[45]
Iron Homeostasis	Deferiprone	Reduction in labile iron pool decreases lipid peroxidation initiation	Clinical and preclinical studies	Indirect (upstream effect)	Does not directly modulate redox systems; systemic effects	[22,56]

These interventions are not specific to the PMRS and primarily act through broad metabolic and redox pathways. Their inclusion reflects conceptual relevance to membrane-associated redox regulation, rather than established PMRS-directed therapeutic targeting. Abbreviations: BBB, blood–brain barrier; CoQ10, coenzyme Q10 (ubiquinone); CYB5R, cytochrome b5 reductase; DMF, dimethyl fumarate; NAD^+^, oxidized nicotinamide adenine dinucleotide; NADH, reduced nicotinamide adenine dinucleotide; NADPH, reduced nicotinamide adenine dinucleotide phosphate; NMN, nicotinamide mononucleotide; NQO1, NAD(P)H quinone dehydrogenase 1; NR, nicotinamide riboside; NRF2, nuclear factor erythroid 2–related factor 2; PMRS, plasma membrane redox system.

## Data Availability

No new data were created or analyzed in this study. Data sharing is not applicable to this article.

## Abbreviations

The following abbreviations are used in this manuscript:

4-HCA	4-hydroxycinnamic acid
4-HNE	4-hydroxynonenal
AA	Arachidonic acid
ADP	Adenosine diphosphate
CD38	Cluster of differentiation 38
CNS	Central nervous system
CoQ	Coenzyme Q
COVID-19	Coronavirus disease 2019
CYB5R	Cytochrome b5 reductase
DHA	Docosahexaenoic acid
DNA	Deoxyribonucleic acid
FSP1	Ferroptosis suppressor protein 1
GPX4	Glutathione peroxidase 4
GSH	Glutathione
IL	Interleukin
MDA	Malondialdehyde
NAD^+^	Nicotinamide adenine dinucleotide
NADH	Nicotinamide adenine dinucleotide (reduced form)
NADPH	Nicotinamide adenine dinucleotide phosphate (reduced form)
ND	Neurodegenerative disorder
NQO1	NAD(P)H:quinone oxidoreductase 1
NRF2	Nuclear factor erythroid 2-related factor 2
PARP-1	Poly(ADP-ribose) polymerase 1
PMRS	Plasma membrane redox system
PUFA	Polyunsaturated fatty acid
ROS	Reactive oxygen species
SARS-CoV-2	Severe acute respiratory syndrome coronavirus 2
xCT	Cystine/glutamate antiporter system Xc^−^
